# ApoA5 knockdown improves whole-body insulin sensitivity in high-fat-fed mice by reducing ectopic lipid content

**DOI:** 10.1194/jlr.M054080

**Published:** 2015-03

**Authors:** João Paulo G. Camporez, Shoichi Kanda, Max C. Petersen, François R. Jornayvaz, Varman T. Samuel, Sanjay Bhanot, Kitt Falk Petersen, Michael J. Jurczak, Gerald I. Shulman

**Affiliations:** * Departments of Internal Medicine Yale University School of Medicine, New Haven, CT; † Cellular and Molecular Physiology, Yale University School of Medicine, New Haven, CT; ** Howard Hughes Medical Institute, Yale University School of Medicine, New Haven, CT; § Isis Pharmaceuticals, Carlsbad, CA

**Keywords:** diacylglycerol, dyslipidemias, insulin signaling, lipase/lipoprotein, apolipoprotein, protein kinase C, lipid uptake, insulin resistance, nonalcoholic fatty liver disease

## Abstract

ApoA5 has a critical role in the regulation of plasma TG concentrations. In order to determine whether ApoA5 also impacts ectopic lipid deposition in liver and skeletal muscle, as well as tissue insulin sensitivity, we treated mice with an antisense oligonucleotide (ASO) to decrease hepatic expression of ApoA5. ASO treatment reduced ApoA5 protein expression in liver by 60–70%. ApoA5 ASO-treated mice displayed approximately 3-fold higher plasma TG concentrations, which were associated with decreased plasma TG clearance. Furthermore, ApoA5 ASO-treated mice fed a high-fat diet (HFD) exhibited reduced liver and skeletal muscle TG uptake and reduced liver and muscle TG and diacylglycerol (DAG) content. HFD-fed ApoA5 ASO-treated mice were protected from HFD-induced insulin resistance, as assessed by hyperinsulinemic-euglycemic clamps. This protection could be attributed to increases in both hepatic and peripheral insulin responsiveness associated with decreased DAG activation of protein kinase C (PKC)-ε and PKCθ in liver and muscle, respectively, and increased insulin-stimulated AKT2 pho­sphory­lation in these tissues. In summary, these studies demonstrate a novel role for ApoA5 as a modulator of susceptibility to diet-induced liver and muscle insulin resistance through regulation of ectopic lipid accumulation in liver and skeletal muscle.

ApoA5 is synthesized by the liver and secreted into the plasma, where it is associated with VLDL, chylomicrons, and HDL particles. Several human and mouse studies have shown that ApoA5 plays an important role in the regulation of plasma TG concentrations. Transgenic mice overexpressing ApoA5 displayed lower levels of plasma TGs compared with WT mice, whereas ApoA5 knockout mice developed hypertriglyceridemia (1). Also, adenoviral overexpression of ApoA5 in mice significantly reduced plasma levels of TGs and cholesterol (2). In humans, ApoA5 single nucleotide polymorphisms have been found to modify plasma TG levels (3–5). ApoA5 gene variants were also observed to influence the risk of myocardial infarction (6). The mechanism by which ApoA5 reduces plasma TG concentrations is not completely understood, although it has been established that ApoA5 activates LPL activity, thus accelerating hydrolysis of TG-rich lipoproteins (7).

Given previous studies that have demonstrated that alterations in LPL activity can alter ectopic lipid deposition in liver and skeletal muscle (8–11), we hypothesized that knockdown of ApoA5 expression in liver would alter ectopic lipid storage in liver and skeletal muscle and insulin action in these tissues. In order to test this hypothesis, we examined the impact of knocking down hepatic ApoA5 expression by targeted antisense oligonucleotide (ASO) treatment on diet-induced hepatic steatosis and liver and muscle insulin resistance in awake mice.

## MATERIALS AND METHODS

### Animals

Male C57BL/6J mice were individually housed under controlled temperature (23°C) and lighting (12:12 h light/dark cycle, lights on at 7:00 AM) with free access to water and food. Mice, at 12 weeks of age, were injected intraperitoneally with control ASO (control) or ApoA5 ASO (ASO) twice weekly at a dose of 50 mg/kg/week for 8 weeks. During the ASO treatment, mice were fed with a regular rodent chow (RC) or a high-fat diet (HFD) (D12492; Research Diets, New Brunswick, NJ). Body composition was assessed by ^1^H magnetic resonance spectroscopy using a Bruker Minispec analyzer (Bruker BioSpin, Billerica, MA). Energy expenditure, respiratory quotient, whole-body oxygen consumption, whole-body carbon dioxide production, locomotor activity, and food intake were measured using a comprehensive lab animal metabolic system (CLAMS; Columbus Instruments, Columbus, OH). Drinking was measured as previously described (12). All experimental procedures were approved by and conducted in accordance with the Institutional Animal Care and Use Committee guidelines of Yale University School of Medicine.

### Plasma measurements

Plasma was collected from 6 h-fasted mice and assayed for LPL activity using a commercially available kit (Cell Biolabs, Inc.). Plasma TG concentrations were measured using a DCL TG reagent (Diagnostic Chemicals). Plasma fatty acids were determined with the NEFA C kit (Wako Pure Chemical Industries). Plasma insulin concentrations were measured by RIA (Millipore). Plasma cytokines were measured using a mouse V-PLEX Proinflammatory Panel 1 (Meso Scale Discovery).

### Tissue lipid measurements

Tissue TGs were extracted using the method of Folch et al. (13) and measured using a DCL TG reagent (Diagnostic Chemicals). For diacylglycerol (DAG) extraction, livers and muscles were homogenized in buffer A [20 mM Tris-HCl (pH 7.4), 1 mM EDTA, 0.25 mM EGTA, and 250 mM sucrose] containing a protease inhibitor mixture (Roche), and samples were centrifuged at 100,000 *g* for 1 h at 4°C. The supernatants containing the cytosolic fraction were collected for DAG measurement and the pellet containing the membrane fraction was resuspended in 700 μl buffer A for DAG analysis. DAG levels were measured by LC/MS/MS as previously described (14). Ceramide was measured as previously described (14). Total cytosolic and membrane DAG and ceramide content are expressed as the sum of individual species. All lipid measurements were made from tissues harvested from 6 h-fasted mice.

### Hyperinsulinemic-euglycemic clamp studies

Hyperinsulinemic-euglycemic clamps were performed as previously described (15). Mice were implanted with jugular venous catheters 7 days before hyperinsulinemic-euglycemic clamps. Basal whole-body glucose turnover was measured by infusing [3-^3^H]glucose (HPLC purified; PerkinElmer Life Sciences) at a rate of 0.05 μCi/min for 120 min into the jugular catheter after a 6 h fast. Following this basal period, hyperinsulinemic-euglycemic clamps were conducted in conscious mice for 140 min with a 4 min primed infusion of insulin (7.14 mU/kg/min) and [3-^3^H]glucose (0.24 μCi/min), followed by a continuous (3 mU/kg/min) infusion of human insulin (Novolin; Novo Nordisk) and [3-^3^H]glucose (0.1 μCi/min), and a variable infusion of 20% dextrose to maintain euglycemia (∼120 mg/dl). After 85 min, a 10 μCi bolus of 2-deoxy-D-[1-^14^C]glucose (PerkinElmer) was injected to estimate insulin-stimulated tissue glucose uptake. Blood for plasma samples was collected from the tail at 0, 25, 45, 65, 80, 90, 100, 110, 120, 130, and 140 min. The tail incision for sample collection was made at least 2 h before the first blood sample was taken to allow for acclimatization, according to standard operating procedures (16). Also, mice received an intravenous albumin-containing solution mimicking artificial plasma, at a rate of 4.2 μl/min, during the insulin-stimulated period of the clamp to compensate for volume loss secondary to blood sampling. Mice were anesthetized with pentobarbital sodium injection (150 mg/kg) at the end of the clamp. All tissues were quickly excised, snap-frozen in liquid nitrogen, and stored at −80°C for subsequent analysis.

### Plasma lipid clearance and tissue uptake

Plasma lipid clearance and tissue uptake were assessed using [^3^H]labeled triolein, as previously described (10). Briefly, mice were implanted with a jugular venous catheter 7 days before the experiment. After overnight fasting, a basal blood sample was collected from the tail and a bolus of 100 μl Intralipid (20%; Abbott Laboratories, North Chicago, IL) conjugated with 10 μCi of [9,10-^3^H(N)]triolein was delivered through the jugular vein. Following this, blood samples were collected at 2, 5, 10, and 15 min from the tail. Plasma and tissue lipids were extracted using the method of Folch et al. (13) and ^3^H radioactivity was measured by scintillation counting. An oral lipid tolerance test was also performed. Following an overnight fast, mice received a gavage of lard (400 μl/mouse) and blood samples were collected at 0, 1, 2, 3, 4, and 6 h for plasma TG determination.

### Liver TG production

In order to determine the rate of liver TG production in mice, blood samples were collected after overnight fasting to determine basal plasma TG levels. After basal blood collection, control and ApoA5 ASO-treated mice were injected intraperitoneally with poloxamer 407 (1 g/kg of body weight; Sigma-Aldrich, USA) to inhibit LPL activity. Blood samples were collected 1, 2, 3, and 4 h after the injection. The VLDL-TG production rate was calculated by the increase in plasma TG level from baseline (17).

### Immunoblotting analysis

For AKT2 phosphorylation, tissues were homogenized in RIPA lysis buffer supplemented with protease inhibitor cocktail (Roche) for protein isolation. Thirty micrograms of proteins from homogenized liver, skeletal muscle, or white adipose tissue (WAT) were separated by 4–12% SDS-PAGE (Invitrogen) and then transferred to polyvinylidene difluoride membranes (Millipore) using a semidry transfer cell (Bio-Rad) for 120 min. For novel protein kinase C (nPKC) translocation in liver and muscle, tissues were homogenized in buffer A [20 mM Tris-HCl (pH 7.4), 1 mM EDTA, 0.25 mM EGTA, 250 mM sucrose, and protease inhibitor cocktail]. Samples were centrifuged at 40,000 rpm at 4°C for 1 h and the supernatants were collected (cytosolic fraction). Membrane pellets were homogenized in buffer B [250 mM Tris-HCl (pH 7.4), 1 mM EDTA, 0.25 mM EGTA, 2% Triton X-100, and protease inhibitor cocktail]. Samples were centrifuged at 40,000 rpm at 4°C for 1 h to obtain the membrane fraction. Thirty micrograms of proteins from cytosolic and membrane fractions were separated by 4–12% SDS-PAGE (Invitrogen) and then transferred to polyvinylidene difluoride membranes (Millipore) using a semidry transfer cell (Bio-Rad) for 120 min. After blockade of nonspecific sites with 5% nonfat dry milk/TBST (10 mM Tris, 100 mM NaCl, and 0.1% Tween-20) solution, membranes were incubated overnight at 4°C with the following primary antibodies: phospho-AKT2^ser474^ (Signalway Antibody, USA), AKT2 (Cell Signaling Technology, Inc., Danvers, MA), PKCε (BD Transduction Laboratories, Lexington, KY), PKCθ (BD Transduction Laboratories), or GAPDH (Santa Cruz Biotechnology, Inc., Santa Cruz, CA). After washing with TBST, membranes were incubated with horseradish peroxidase-conjugated anti-rabbit, anti-mouse, or anti-goat antibody. Membranes were thoroughly washed, and immune complexes were detected using an ECL system (Thermo Scientific, USA) and developed on photographic films. Signals on the immunoblot were quantified by optical densitometry (Image J Software, Bethesda, MD).

### Total RNA preparation and real-time quantitative PCR analysis

Total RNA was extracted from snap-frozen liver and WAT using the RNeasy kit (Qiagen). One microgram of RNA was reverse-transcribed to cDNA using the Quantitect RT kit (Qiagen) as per the manufacturer’s protocol. The abundance of transcripts was assessed by real-time PCR on a 7500 Fast real-time PCR system (Applied Biosystems) with a SYBR Green detection system. Primer sequences were as follows: ApoA5 (forward, GGCGAGTTCTGCCGTAGGA; reverse, CCCAACCCCATCAAATGTGA), ApoC2 (forward, CATGGGGTCTCGGTTCTTCC; reverse, ATGCCTGCGTAAGTGCTCAT), ApoC3 (forward, CCACAGA­AGGCT­TGGGAC­TC; reverse, AGGAACAGGCACATCTGCAA), HL (forward, GACATC­GG­CGAGTTGATCCT; reverse, GCAAAG­CACACCCTC­CTTTAC), SREBP1c (forward, CAGCTCAGAGCCGTGGTGA; reverse, TTGATAGAAGACCGGTAGCGC), PPARγ (forward, ATGCCAAAAATATCCCTGGTTTC; reverse, GGAGGCCAGCATGGTGTAGA), FAS (forward, GCTGCGGA­AACT­TC­AGGAAAT; reverse, AGAG­ACGT­GTCAC­TCCTGG­ACT), DGAT2 (forward, ACT­CTGG­AGGTTG­GC­ACCAT; reverse, GGGTGTGGCT­CAGGAGGAT), and ACC1 (forward, GCCATT­GGT­ATT­GGGGCTTAC; reverse, CCCGAC­CAAG­GACTTTGTTG). GAPDH (for­ward, CTCCACTC­ACG­GCAAA­TTCA; reverse, ATG­GGCTTC­CCGT­TGATGA) was used as housekeeping gene and data were normalized for the efficiency of amplification, as determined by a standard curve included on each run.

### Statistical analysis

All data are expressed as mean ± SEM. Results were assessed using two-tailed unpaired Student’s *t*-test or two-way ANOVA (GraphPad Prism 5, La Jolla, CA). A *P* value less than 0.05 was considered significant.

## RESULTS

### ApoA5 knockdown decreased body fat in HFD-fed mice

**Fig. 1.**
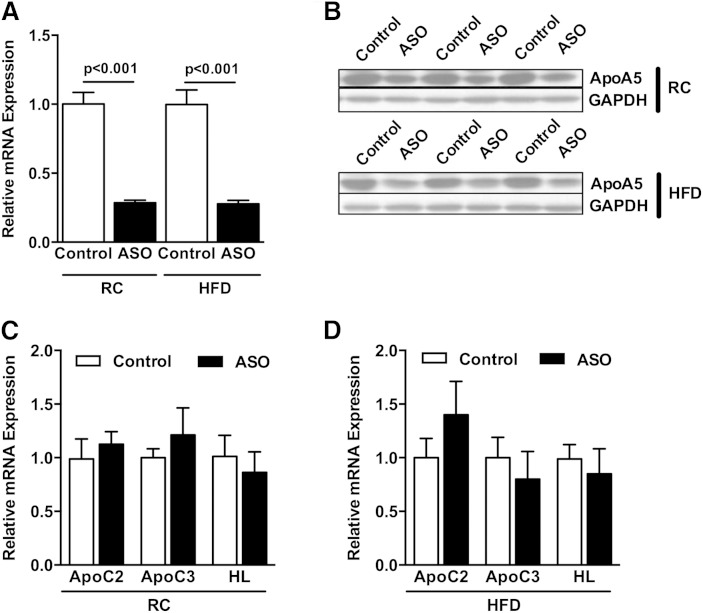
ApoA5 ASO treatment reduced ApoA5 mRNA and protein expression. A: Liver ApoA5 mRNA expression from vehicle- and ASO-treated mice fed RC or HFD. B: Liver ApoA5 protein expression from vehicle- and ASO-treated mice fed RC or HFD. C: Liver ApoC2, ApoC3, and HL mRNA expression from vehicle- and ASO-treated mice fed RC. D: Liver ApoC2, ApoC3, and HL mRNA expression from vehicle- and ASO-treated mice fed a HFD. Data are represented as mean ± SEM (n = 8 per group).

To assess the potential role of ApoA5 on whole-body energy metabolism and lipid-induced insulin resistance, we fed mice RC or HFD and decreased hepatic ApoA5 expression by ASO treatment for 8 weeks. The chief advantage of this approach is that ASO treatment effectively reduces gene expression primarily in liver and WAT, and avoids any compensatory developmental effects associated with gene-knockout mouse models. As shown in **Fig. 1A**, ApoA5 ASO treatment effectively reduced ApoA5 mRNA expression in liver by ∼70% in both RC- and HFD-fed mice. Similarly, ApoA5 protein levels were reduced by ∼60% in ASO-treated mice (Fig. 1B). Consistent with other studies showing that ApoA5 modulation affects plasma TG levels, ApoA5 ASO-treated mice displayed ∼3-fold higher plasma TG concentrations compared with control mice (**Table 1**).

**TABLE 1.** Basal characterization of animals

	RC		HFD	
	Control	ASO	Control	ASO
Body weight (g)	29.9 ± 0.8	31.4 ± 0.9	41.6 ± 1.7	38.7 ± 1.3
Body fat (g)	2.9 ± 0.1	3.0 ± 0.1	12.9 ± 1.7	7.4 ± 1.1*^a^*
Fasting glucose (mg/dl)	110 ± 6.9	113 ± 4.3	135 ± 4.9	133 ± 6.9
Fasting insulin (μU/ml)	9.1 ± 0.8	8.7 ± 0.9	22.0 ± 2.3	13.3 ± 1.3*^b^*
Fasting NEFA (mmol/l)	0.43 ± 0.06	0.75 ± 0.07*^a^*	0.39 ± 0.01	0.65 ± 0.05*^a^*
Fasting plasma TG (mg/dl)	54.1 ± 2.0	168.2 ± 17.5*^b^*	62.9 ± 2.6	153.8 ± 12.1*^b^*
Whole-body oxygen consumption (ml/kg/hr)	2,920 ± 94	2935 ± 82	3140 ± 74	3108 ± 131
Whole-body carbon dioxide production (ml/kg/hr)	2,661 ± 99	2,657 ± 84	2,463 ± 68	2,389 ± 109
Energy expenditure (kcal/kg/hr)	14.3 ± 0.4	14.4 ± 0.4	14.9 ± 0.3	14.8 ± 0.6
Caloric intake (kcal/kg/hr)	17.3 ± 0.7	16.8 ± 1.0	15.2 ± 0.4	15.0 ± 0.3
Drinking (ml/kg/hr)	1.1 ± 0.2	1.1 ± 0.1	1.0 ± 0.1	0.9 ± 0.1
Activity (counts/hr)	171 ± 22	145 ± 17	168 ± 18	141 ± 5

Control and ApoA5 ASO-treated mice fed RC or a HFD for 8 weeks. Data are expressed as mean values ± SEM.

a *P* < 0.05.

b *P* < 0.01 by two-way ANOVA compared with control versus ASO in each diet.

To rule out compensatory effects of ApoA5 knockdown on other regulators of plasma TG levels, relative mRNA expression of ApoC2, ApoC3, and HL was evaluated. We observed no differences in hepatic expression of these genes in ApoA5 ASO-treated mice fed either RC or HFD (Fig. 1C, D).

In comparison to chow-fed mice, high-fat feeding increased body weight and adiposity in both control and ApoA5 ASO-treated mice (Table 1). ApoA5 ASO treatment did not modify the weight gain induced by HFD, although it reduced adiposity compared with control mice (Table 1). In order to examine whether changes in energy balance were responsible for the reduced adiposity observed in ASO-treated mice, whole-body metabolism was monitored by metabolic cage in RC- and HFD-fed mice. ApoA5 ASO treatment did not modify whole-body O_2_ consumption and energy expenditure compared with control mice (Table 1) in either RC- or HFD-fed mice. Caloric intake, drinking, and ambulatory activity were also not different between groups (Table 1).

### ApoA5 knockdown decreased plasma lipid clearance and tissue uptake

**Fig. 2.**
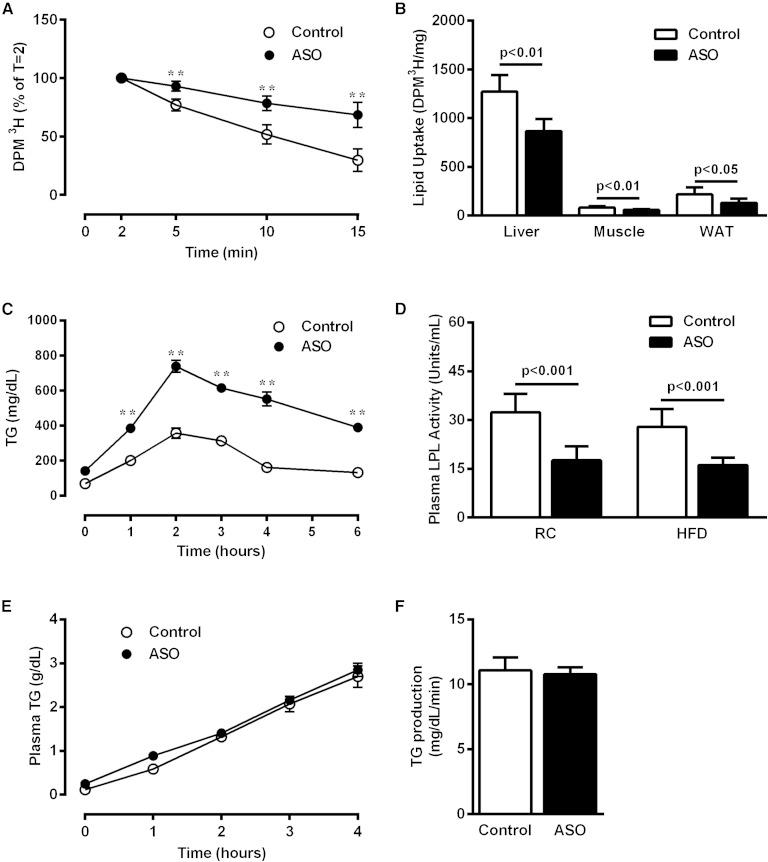
ApoA5 knockdown decreased plasma lipid clearance. A: Plasma lipid clearance assayed by an iv bolus of ^3^H-labeled triolein. B: Tissue lipid uptake measured 15 min after an iv bolus of ^3^H-labeled triolein. C: Plasma TG excursion in overnight-fasted mice after a lard gavage (400 μl/mouse). D: Whole plasma LPL activity in mice fasted 6 h. E: Plasma TG levels in overnight-fasted mice after an intraperitoneal injection of poloxamer. F: Liver TG production rate in overnight-fasted mice. Data are represented as mean ± SEM (n = 8 per group). **P < 0.01 compared with vehicle-treated mice.

Available evidence suggests that ApoA5 activates LPL, leading to increased TG-rich lipoprotein hydrolysis and concomitant TG removal from plasma. Therefore, the increased plasma TG levels observed in ApoA5 ASO-treated mice might owe to reduced LPL activity. Indeed, ApoA5 ASO-treated mice displayed reduced plasma lipid clearance after a bolus injection of Intralipid compared with control mice (**Fig. 2A**). Consistent with these findings, ApoA5 ASO-treated mice exhibited reduced hepatic, muscle, and WAT lipid uptake (Fig. 2B). To determine whether the route of lipid administration influenced this effect, an oral lipid tolerance test was performed. After lard gavage, plasma lipid clearance was reduced in ApoA5 ASO-treated mice (Fig. 2C), consistent with the intravenous Intralipid injection study. Plasma LPL activity was measured and found to be decreased in ApoA5 ASO-treated mice, consistent with the hypothesis that LPL mediates ApoA5 effects on plasma TG (Fig. 2D).

In order to examine whether hepatic VLDL-TG production contributes to the increased plasma TG seen in ApoA5 ASO-treated mice, we evaluated rates of liver VLDL-TG production. There was no difference in hepatic VLDL-TG production rate between groups (Fig. 2E, F).

**Fig. 3.**
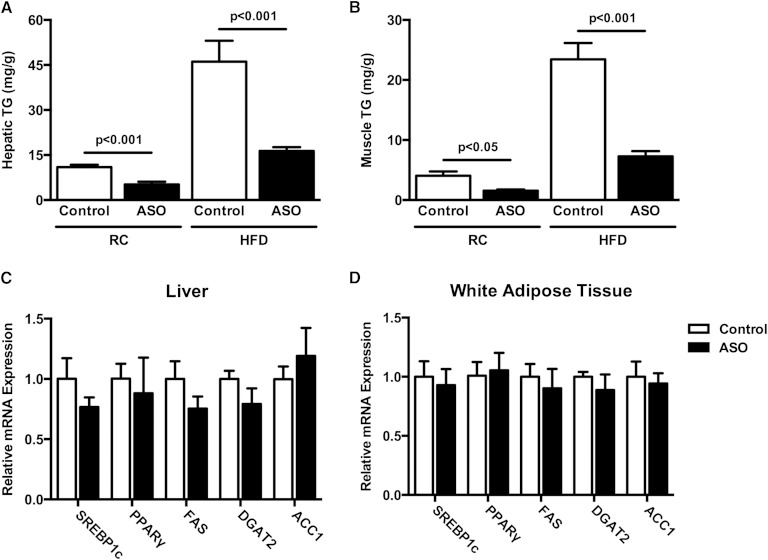
ApoA5 knockdown reduced hepatic and muscle TG content. A: Hepatic TG content in mice fasted 6 h. B: Muscle TG content in mice fasted 6 h. C: Relative mRNA expression of lipid synthetic enzymes in liver. D: Relative mRNA expression of lipid synthesis enzymes in WAT. Data are represented as mean ± SEM (n = 8 per group).

The reduced plasma TG clearance observed in ApoA5 ASO-treated mice led us to hypothesize that these mice would exhibit decreased tissue lipid content. To test this hypothesis, we measured TG content in liver and muscle of control and ApoA5 ASO-treated mice. In both liver and skeletal muscle, ApoA5 ASO-treated mice displayed reduced TG content compared with control mice (**Fig. 3A, B**). This effect was observed in both RC- and HFD-fed mice, and was not associated with alterations in mRNA expression of the key lipid synthetic enzymes, SREBP1c, PPARγ, FAS, DGAT2, or ACC1, in either liver or WAT (Fig. 3C, D).

### ApoA5 knockdown increased whole-body insulin sensitivity in HFD-fed mice

**Fig. 4.**
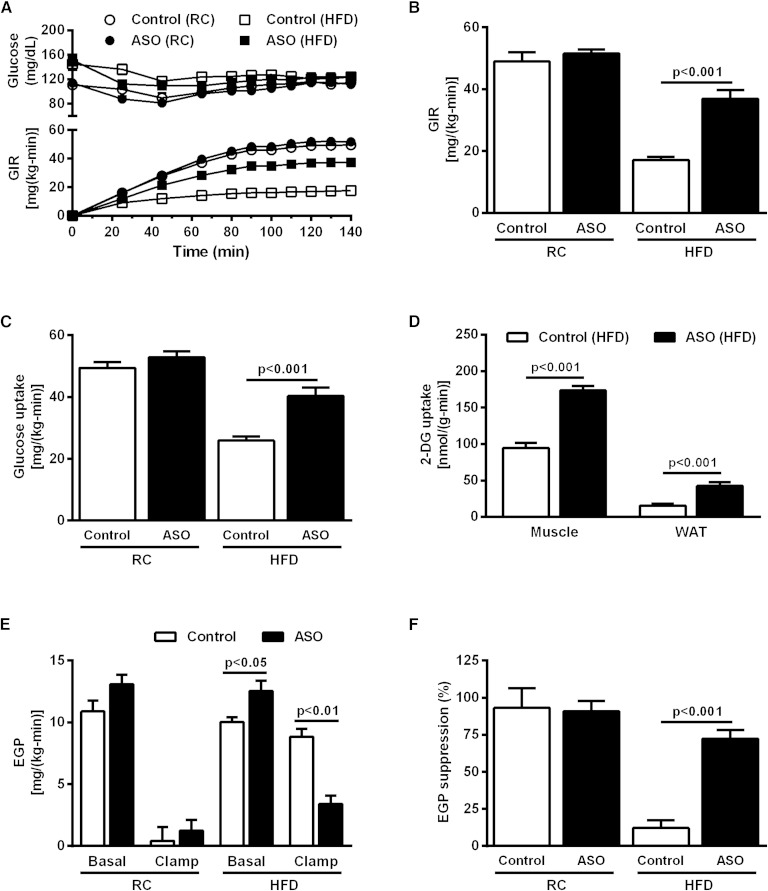
ApoA5 knockdown increased whole-body insulin sensitivity in HFD-fed mice. A: Blood glucose and GIR during hyperinsulinemic-euglycemic clamp. B: GIR during the steady-state period (last 40 minutes) of the hyperinsulinemic-euglycemic clamp. C: Whole-body glucose uptake during the steady-state period of the hyperinsulinemic-euglycemic clamp. D: Muscle and adipose tissue 2-deoxyglucose uptake during the hyperinsulinemic-euglycemic clamp. E: EGP measured in the basal period and during the hyperinsulinemic-euglycemic clamp. F: EGP suppression (percent basal) during hyperinsulinemic-euglycemic clamp. Data are represented as mean ± SEM (n = 8 per group).

Given the effect of ApoA5 knockdown in ectopic lipid content in liver and muscle and the strong relationship between ectopic lipid content and insulin resistance in these tissues (15, 18, 19), we performed hyperinsulinemic-euglycemic clamp studies combined with ^3^H/^14^C-labeled glucose infusions in order to determine whether the reduced ectopic lipid content seen in ApoA5 ASO-treated mice was associated with protection from lipid-induced insulin resistance. There was no difference in whole-body insulin sensitivity between control and ApoA5 ASO-treated mice fed a RC diet (**Fig. 4A, B**). HFD-fed control mice were insulin resistant, as manifested by a ∼65% reduction in glucose infusion rate (GIR) required to maintain euglycemia during the hyperinsulinemic-euglycemic clamp compared with RC-fed mice (Fig. 4A, B). However, ApoA5 ASO treatment abrogated this effect; ApoA5 ASO-treated mice fed HFD displayed preserved whole-body insulin sensitivity, as reflected by a more than 2-fold increase in GIR required to maintain euglycemia during the hyperinsulinemic-euglycemic clamp compared with HFD-fed control mice (Fig. 4A, B). The protection from HFD-induced insulin resistance in ApoA5 ASO-treated mice could be partially attributed to enhanced peripheral insulin sensitivity, as evidenced by increased peripheral glucose uptake (Fig. 4C) and increased 2-deoxyglucose uptake in skeletal muscle and WAT (Fig. 4D). Moreover, ApoA5 ASO-treated mice also exhibited protection from HFD-induced hepatic insulin resistance, as reflected by lower rates of endogenous glucose production (EGP) during the hyperinsulinemic-euglycemic clamp compared with control HFD mice (Fig. 4E), reflecting preserved suppression of hepatic glucose production by insulin in the ApoA5 ASO-treated mice (Fig. 4F).

### ApoA5 knockdown reduced liver and muscle DAG content and nPKC translocation

In order to understand the mechanism by which ApoA5 ASO-treated mice were protected from lipid-induced liver and muscle insulin resistance, we assessed DAG and ceramide content, nPKC translocation, and insulin-stimulated AKT2 phosphorylation in these tissues.

**Fig. 5.**
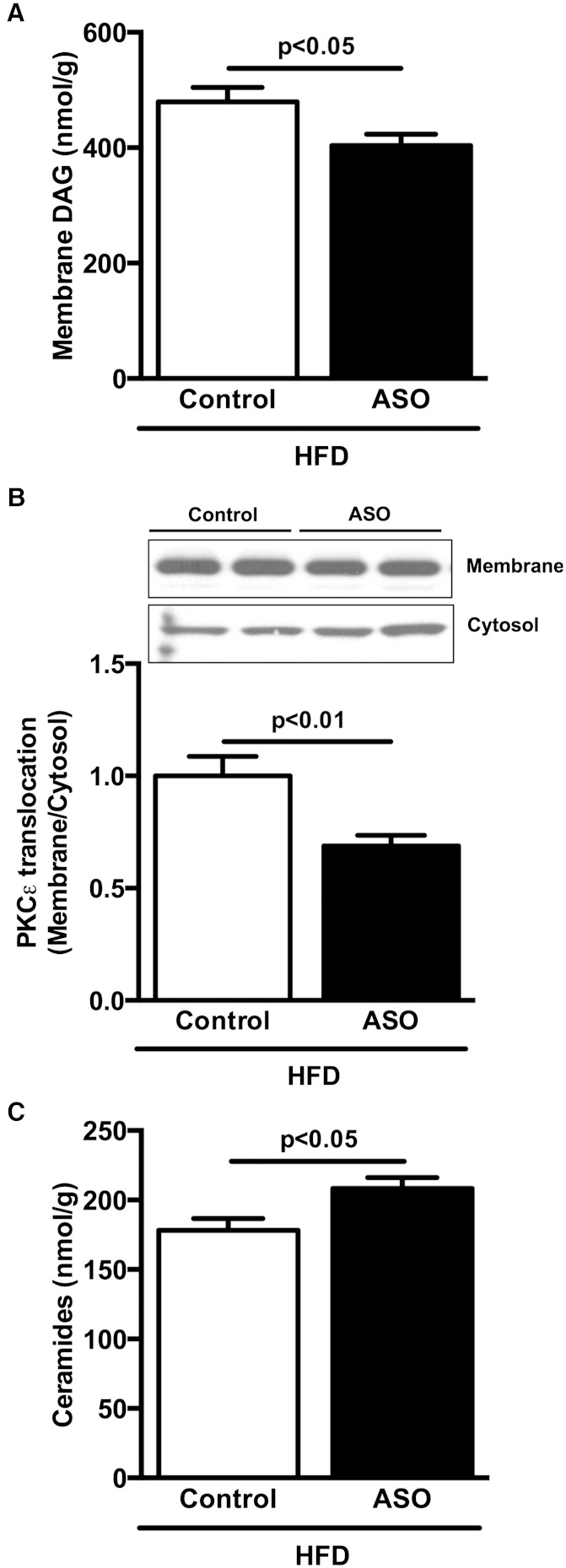
ApoA5 knockdown reduced hepatic membrane DAG content and PKCε activation. A: Hepatic membrane DAG content in mice fasted 6 h. B: Hepatic PKCε membrane translocation in mice fasted 6 h. C: Hepatic ceramide content in mice fasted 6 h. Data are represented as mean ± SEM (n = 8 per group).

In HFD-fed mice, ApoA5 ASO treatment reduced hepatic DAG content compared with control mice (**Fig. 5A**). Because DAG-mediated increases in PKCε activity have been causally implicated in hepatic insulin resistance, we assessed membrane translocation of PKCε in liver. In accordance with the observed reductions in DAG content, ApoA5 ASO-treated mice displayed reduced PKCε membrane translocation (Fig. 5B). In contrast, liver ceramide content was increased in ApoA5 ASO-treated mice compared with control ASO-treated mice despite protection from lipid-induced hepatic insulin resistance in these mice (Fig. 5C).

**Fig. 6.**
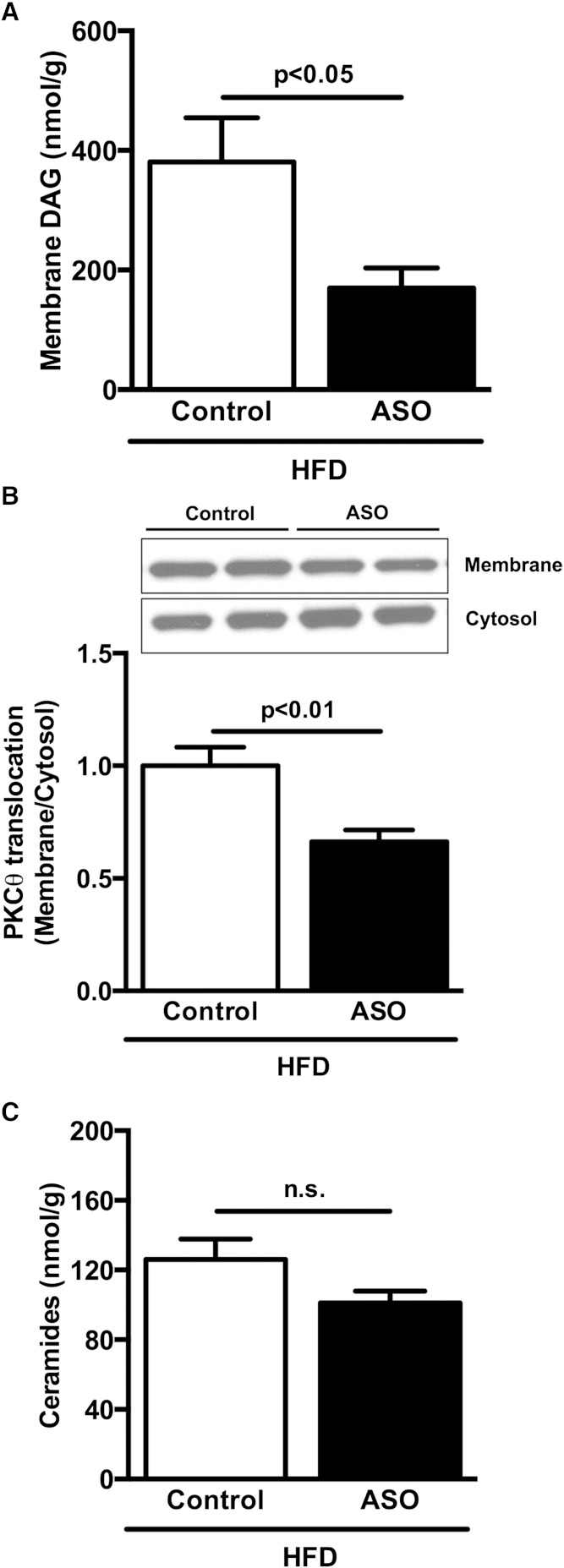
ApoA5 knockdown reduced muscle membrane DAG content and PKCθ activation. A: Muscle membrane DAG content in mice fasted 6 h. B: Muscle PKCθ membrane translocation in mice fasted 6 h. C: Muscle ceramide content in mice fasted 6 h. Data are represented as mean ± SEM (n = 8 per group).

ApoA5 ASO-treated mice fed a HFD also displayed reduced DAG content in skeletal muscle compared with control mice (**Fig. 6A**). Consistent with reduced muscle DAG content, ApoA5 ASO-treated mice displayed reduced PKCθ membrane translocation compared with control mice (Fig. 6B). Muscle ceramide content was not different between groups (Fig. 6C).

**Fig. 7.**
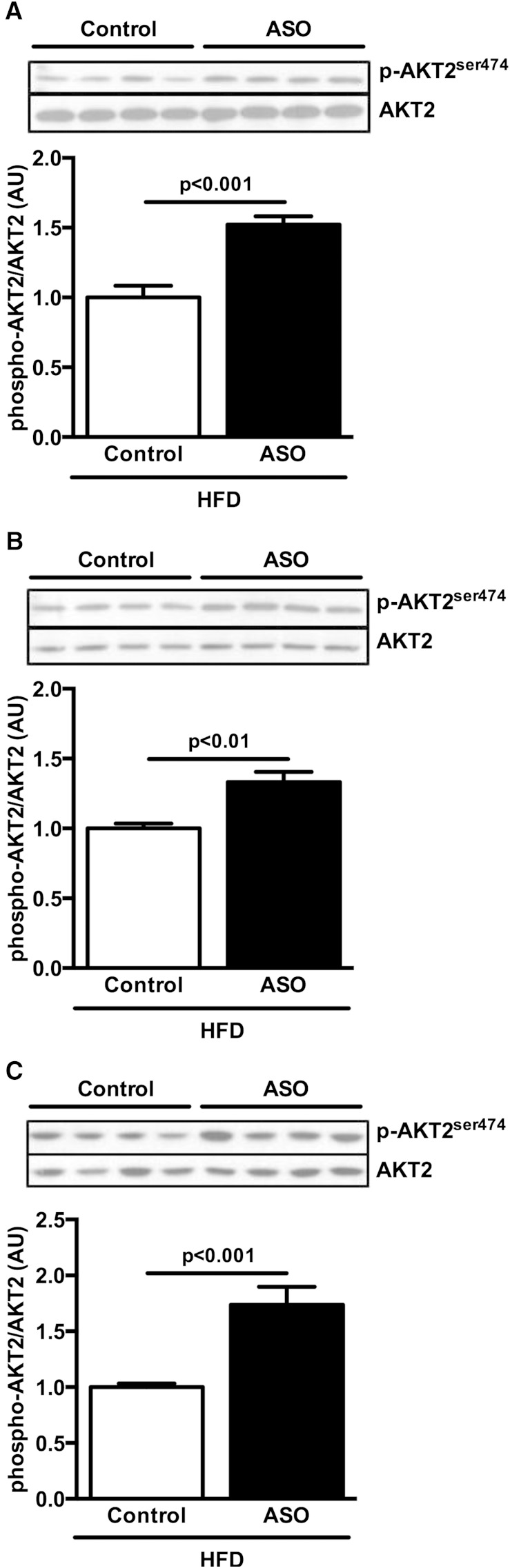
ApoA5 knockdown increased insulin signaling. A):Liver postclamp AKT2 phosphorylation. B: Muscle postclamp AKT2 phosphorylation. C: WAT postclamp AKT2 phosphorylation. Data are represented as mean ± SEM (n = 8 per group).

Finally, the preserved insulin sensitivity in ApoA5 ASO-treated HFD-fed mice was accompanied by increases in insulin-stimulated AKT2 phosphorylation in liver (**Fig. 7A**), skeletal muscle (Fig. 7B), and WAT (Fig. 7C).

### ApoA5 ASO-treated mice display increased plasma cytokines

In order to examine other potential mechanisms for the improved insulin sensitivity observed in ApoA5 ASO-treated mice, plasma cytokine concentrations were evaluated. Unexpectedly, ApoA5 ASO-treated mice fed HFD displayed increased plasma IL-2, IL-6, IL-10, KC/GRO, and TNF-α levels (**Table 2**), indicating that reduced cytokine signaling does not explain the improved insulin sensitivity in ApoA5 ASO-treated HFD-fed mice.

**TABLE 2.** Plasma cytokines concentration in HFD-fed mice

	Control	ASO
INFγ (pg/ml)	0.28 ± 0.05	0.35 ± 0.07
IL-1β (pg/ml)	1.15 ± 0.10	1.19 ± 0.07
IL-10 (pg/ml)	10.7 ± 1.06	37.0 ± 8.46*^b^*
IL-12p70 (pg/ml)	16.6 ± 0.58	16.2 ± 0.76
IL-2 (pg/ml)	0.48 ± 0.09	1.19 ± 0.19*^b^*
IL-4 (pg/ml)	1.76 ± 0.07	1.72 ± 0.08
IL-5 (pg/ml)	2.82 ± 0.24	3.28 ± 0.25
IL-6 (pg/ml)	32.7 ± 6.27	149.8 ± 44.7*^a^*
KC/GRO (pg/ml)	142.5 ± 13.9	217.7 ± 22.8*^a^*
TNF-α (pg/ml)	11.5 ± 1.10	20.1 ± 1.47*^c^*

Control and ApoA5 ASO treated mice fed a HFD for 8 weeks. Data are expressed as mean values ± SEM.

a *P* < 0.05.

b *P* < 0.01.

c *P* < 0.001 by unpaired *t*-test compared with control mice.

## DISCUSSION

In this study, we examined the impact of ApoA5 ASO treatment on ectopic lipid deposition in liver and skeletal muscle, as well as insulin action in these tissues. We found that knockdown of hepatic ApoA5 expression by ASO treatment decreased plasma TG clearance and reduced tissue lipid uptake. This reduced tissue lipid uptake translated into reduced ectopic lipid (TG/DAG) accumulation in liver and muscle, decreased nPKC activation, and protection from HFD-induced liver and muscle insulin resistance.

It has been suggested that the effect of ApoA5 on lipid metabolism is mediated by modulation of LPL activity, with several studies demonstrating evidence for this hypothesis (7, 20, 21). Fruchart-Najib et al. (7) showed that mice overexpressing human ApoA5 exhibited higher plasma LPL activity associated with increased plasma ^3^H-VLDL clearance. Merkel et al. (20) also showed that ApoA5 transgenic mice displayed increased catabolism of VLDL and chylomicrons due to their increased plasma hydrolysis by LPL. They elegantly demonstrated, using transgenic expression of human LPL in ApoA5 knockout mice and ApoA5 overexpression in LPL deficient mice, that increased LPL activity normalized hypertriglyceridemia in ApoA5 knockout mice, while ApoA5 overexpression affected plasma TG levels only slightly when LPL activity was reduced (20). Consistent with these data, we observed increased plasma TG excursions in ApoA5 ASO-treated mice by both oral and intravenous lipid challenge. This observation was associated with reduced tissue-specific lipid uptake (liver, muscle, and WAT) and reduced plasma LPL activity, confirming that ApoA5 modulates plasma TG levels, in part, by increasing LPL activity.

It was particularly interesting to note that hepatic lipid uptake was reduced in ApoA5 ASO-treated mice. Liver lipid uptake is much less dependent on LPL activity compared with skeletal muscle and WAT, because the hydrolysis and clearance of TG-rich lipoproteins by the liver is mediated by HL and by endocytic receptors such as LDL receptor (LDLR), LDLR-related protein 1 (LRP1), and heparan sulfate proteoglycans (HSPGs) such as syndecan-1 (SDC1). Of note, our data are consistent with another recent report, which found reduced plasma lipid clearance in ApoA5 knockout mice (22). ApoA5 has been suggested to promote hepatic clearance of TG-rich lipoproteins by acting as a ligand for receptor-mediated endocytosis, which may explain these observations. Indeed, it was recently shown that ApoA5 mediates hepatic lipoprotein clearance by facilitating the binding of TG-rich lipoproteins to hepatic HSPG (23).

ApoA5 has received considerable attention for its potentially beneficial effects on atherosclerotic risk factors, especially plasma TGs (23, 24). However, the potential role of ApoA5 in ectopic lipid deposition and whole-body glucose metabolism and insulin sensitivity has not yet been well-studied. The hypertriglyceridemia observed after ApoA5 knockdown might lead one to hypothesize that ApoA5 knockdown would diminish tissue insulin sensitivity. Yet ApoA5 ASO-treated mice displayed increased whole-body insulin sensitivity after fat-feeding owing, most likely, to reduced liver and muscle lipid uptake. This finding of improved insulin sensitivity in HFD-fed ApoA5 ASO-treated mice was associated with decreased hepatic and skeletal muscle lipid content (in particular, DAG) leading to reduced PKCε and PKCθ activation in liver and skeletal muscle, respectively. These data are consistent with previous studies, which found that reductions in novel PKC activation are associated with improved insulin sensitivity in liver and skeletal muscle (25–27), and that increased novel PKC activation is associated with lipid-induced insulin resistance in these tissues (15, 28, 29).

Despite profound effects on ectopic lipid accumulation and tissue insulin sensitivity, we observed no effect of hepatic ApoA5 knockdown on food intake, body weight, or energy expenditure. These data are in contrast to a recent study using ApoA5 knockout mice which displayed increased body weight and reduced insulin sensitivity associated with increased hepatic TG content, despite reduced tissue lipid uptake when fed a HFD (22). One potential explanation for these conflicting results is that, in contrast to our ApoA5 ASO-treated mice, the ApoA5 knockout mice were hyperphagic. Caloric oversupply in ApoA5 knockout mice may have overwhelmed the protective effects of reduced ApoA5 expression on tissue lipid uptake. This might also reflect differences in the extent and possibly the duration of ApoA5 deficiency between mouse models. ApoA5 knockout mice display complete absence of ApoA5 protein synthesis and secretion into the plasma from birth, while ApoA5 ASO-treated mice exhibit an ∼70% reduction in hepatic ApoA5 protein expression for several weeks during adulthood. Because administration of ApoA5 is sufficient to decrease food intake (22), the residual ApoA5 produced in ASO-treated mice might be sufficient to prevent the hyperphagia of ApoA5 knockout mice.

In summary, the present study demonstrates that reducing hepatic ApoA5 expression in mice by ASO treatment reduces LPL activity leading to hypertriglyceridemia and decreased lipid uptake by liver, muscle, and WAT. This reduction in lipid uptake is associated with reduced liver and muscle TG/DAG content and protection from lipid-induced insulin resistance in these tissues. Taken together these data demonstrate a novel role for ApoA5 as a modulator of susceptibility to liver and muscle insulin resistance through regulation of ectopic lipid accumulation in liver and skeletal muscle.
